# *Trichospermum lessertianum* comb. n., the correct name for the Cuban species of *Trichospermum* (Malvaceae, Grewioideae) also found in Mexico and Central America

**DOI:** 10.3897/phytokeys.2.731

**Published:** 2011-02-11

**Authors:** Laurence J. Dorr

**Affiliations:** Department of Botany, National Museum of Natural History, MRC–166, Smithsonian Institution, P.O. Box 37012, Washington, D.C. 20013–7012, U.S.A.

**Keywords:** *Belotia*, Cuba, Grewioideae, Malvaceae, Mexico, new combination, Tiliaceae, *Trichospermum*

## Abstract

The correct name for the Cuban species of Trichospermum Bl. (Malvaceae: Grewioideae) also found in Mexico and Central America is Trichospermum lessertianum (Hochr.) Dorr, **comb. n.** The name Trichospermum mexicanum (DC.) Baill., incorrectly applied to this Cuban species, should be restricted to a species endemic to western and southern Mexico.

## Introduction

Trichospermum Bl. (Malvaceae: Grewioideae, or Tiliaceae) is a genus of ca. 40 species found in tropical America, Asia, and the Pacific (Kostermans 1962, 1972). Belotia A. Rich., a generic synonym of Trichospermum, was described from Cuba. Misinterpretations of the legitimacy and identity of its generitype, Belotia grewiifolia A. Rich., have led authors treating Trichospermum (or Belotia) for various floras and revisions to adopt species names that are incorrect. Sorting out this confusion requires determining where Belotia was first published (there are three competing publications); demonstrating that Belotia grewiifolia was nomenclaturally superfluous when published; and establishing the identity of the name, Grewia mexicana DC., that should have been adopted as the generitype of Belotia.

Achille Richard published Belotia in three different works that appeared in the 1840s; in volume 10 of Ramón de la Sagra’s *Historia física, política y natural de la isla de Cuba* (Richard 1845: 82; see also Stafleu and Cowan 1983: no. 10.000), in an unnumbered volume of a French edition of the same work (Richard 1841: 207; see also Stafleu and Cowan 1983: nos. 9150, 10.002), and in the second volume of Charles d’Orbigny’s *Dictionnaire universel d’histoire naturelle* (Richard 1842: 539; see also Stafleu and Cowan 1981: no. 7096; Evenhuis 1990). The volumes of the French version of de la Sagra’s *Historia*, at least, were issued in parts (livraisons) and these parts were distributed well before the publication dates given on the volumes as a whole (Brizicky 1962: 84–86; see also Stafleu and Cowan 1983: no. 9150). While the title pages of both the French and Spanish volumes of de la Sagra’s *Historia* that include Belotia have the year 1845, Brizicky (1962: 84–86) determined that the description of Belotia in the French edition actually appeared in a part (livraison) issued in 1841. The strongest evidence for this is the review of Richard’s contribution to Cuban botany published by Grisebach (1842), which established that the livraison containing Belotia was available by the end of 1841. This is the publication date accepted by Stafleu and Cowan (1983: nos. 9150, 10.002). The publication of the second volume of d’Orbigny’s *Dictionnaire*, which included a description of Belotia, followed in 1842. Stafleu and Cowan (1981: no. 7096) dated this volume 30 July 1842, but Evenhuis (1990) subsequently presented evidence that 20 June 1842 is the latest date at which the livraison containing a description of Belotia could have appeared. (The earliest possible, but not probable, date for the publication of this livraison is 24 January 1842). The description of Belotia in the *Dictionnaire* cites the paging and plate number (albeit incorrect) of the Spanish edition of de la Sagra’s *Historia*, which implies that the latter was typeset but presumably not yet distributed. No other evidence has surfaced indicating that the publication of Belotia in the Spanish edition of de la Sagra’s *Historia* was earlier than the date on the title page (i.e., 1845; see also Stafleu and Cowan 1983: no. 10.000) and without proof establishing some other date, the one appearing in the printed matter must be accepted as correct (see McNeill et al. 2006: Art. 31.1).

The fact that Belotia was first published in the French edition of de la Sagra’s *Historia* has nomenclatural implications for the legitimacy of its generitype, Belotia grewiifolia. In the French edition, Belotia grewiifolia (Richard 1841: 209) is a superfluous renaming of Grewia mexicana DC. (1824: 510) as the latter name was placed in synonymy and is the name that should have been adopted (McNeill et al. 2006: Art. 52). Later, in both d’Orbigny’s *Dictionnaire* and in the Spanish edition of de la Sagra’s *Historia*, Richard equivocated with respect to this synonymy. In the former instance, he (1842: 540) wrote “Belotia greviæfolia Rich. (*Fl. Cubens* p. 82, t. 22), qui est probablement le Grewia mexicana DC.” and in the latter (1845: 83) he cited Grewia mexicana in synonymy with a question mark. Sprague (1921), who revised the genus Belotia, recognized that the Mexican and Cuban species differed, but he failed to appreciate that Belotia grewiifolia was an illegitimate name and he used this name for one of two species he recognized from Cuba. He also confused Belotia mexicana (DC.) K. Schum. (basionym Grewia mexicana) with yet a different species occurring in Mexico and Central America. Bullock (1939) continued to use the illegitimate name Belotia grewiifolia for a species from Cuba, but expanded his concept of this taxon’s range to include Central America. He also continued to confuse the identity of Belotia mexicana. Farr et al. (1979: 91) and Rodríguez Fuentes (2000: 31) began to clarify the nomenclatural confusion by recognizing that Trichospermum grewiifolia is illegitimate, but the former did so while citing a place of publication (d’Orbigny’s *Dictionnaire*) that if in fact had been the earliest publication would have resulted in the legitimate publication of the name (see McNeill et al. 2006: Art. 52, Note 1, Ex. 12) and the latter although citing the earliest place of publication failed to realize that Grewia mexicana is not conspecific with the Cuban species of Trichospermum.

Although the plate accompanying the description of Belotia grewiifolia is numbered tab. 21, the protologues of both Spanish and French versions of de la Sagra’s *Historia* incorrectly cite tab. 22, which is a plate illustrating Triumfetta grossulariifolia A. Rich. (Malvaceae: Grewioideae). The plate caption for Belotia grewiifolia in the French (Richard 1841: 211), but not the Spanish version (Richard 1845: 84), however, is correctly labeled tab. 21. Although the plates today invariably are bound separately from the text in a folio volume, text and plates originally were probably available at the same time as each livraison of the French version, at least, of de la Sagra’s Historia was projected to contain four folio plates accompanied by four sheets of text in octavo (see Stafleu and Cowan 1983: no. 9150). As early as March–June 1842 there is a published reference (Endlicher 1842: 108; “Belotia *A. Richard Flor. cub. 207. t. 22*”) to the French text of Belotia grewiifolia and its plate (albeit misnumbered).

The type of Grewia mexicana agrees well with the species treated as Trichospermum insigne (Baill.) Kosterm. in the *Flora Nova–Galiciana* (Fryxell 2001), which has broadly ovate leaf blades with acute apices and a dense and evenly stellate–tomentum below, flowers with sepals and petals ca. 10 mm long, and capsules 16–18 × 24–28 mm. This Mexican species is very distinct from the material of Trichospermum collected in Cuba, which has ovate leaf blades with acuminate to long acuminate apices and a sparse stellate–tomentum below, flowers with sepals and petals 4–6 mm long, and capsules 8–10 × 10–12 mm. A new combination for the Cuban species of Trichospermum is necessary as the earliest available epithet belongs to a species of Belotia. The name Trichospermum mexicanum, misapplied to the Cuban species, is here considered to apply to a species endemic to western and southern Mexico that is frequently but incorrectly cited as Trichospermum insigne.

## Taxonomic summary

### Trichospermum lessertianum

(Hochr.) Dorr comb. nov.

urn:lsid:ipni.org:names:77109528-1

Belotia lessertiana  Hochr., Annuaire Conserv. Jard Bot. Genève 18–19: 90. 1914, as “Lessertiana.” Belotia grewiifolia var. *lessertiana* (Hochr.) Vict., Contr. Inst. Bot. Univ. Montréal 63: 13. 1948. TYPE: CUBA. La Havane, s.d. (fl), Delessert s.n. (holotype: G-DEL; isotypes: K, NY! [00084148], P). (Basionym)
Belotia caribaea  Sprague, Bull. Misc. Inform. Kew 1921(7): 276. 1921. Trichospermum caribaeum (Sprague) Kosterm., Reinwardtia 6(3): 278. 1962. TYPE: ST. LUCIA, s.d. (fl, fr), Anderson s.n. (holotype: K! [K000381875]).
Belotia reticulata  Sprague, Bull. Misc. Inform. Kew 1921(7): 277. 1921. Trichospermum reticulatum (Sprague) Kosterm., Reinwardtia 6(3): 279. 1962. TYPE: NICARAGUA. Chontales, *Seemann 11* (holotype: K).
Belotia campbellii  Sprague, Bull. Misc. Inform. Kew 1921(7): 277. 1921, as “Campbellii.” TYPE: BELIZE. Seven Hills Estate, s.d. (fl, fr), *E.J.F. Campbell 75* (holotype: K! [K000381880]; isotype: F).
Belotia tabascana  Sprague, Bull. Misc. Inform. Kew 1921(7): 278. 1921. Trichospermum tabascanum (Sprague) Kosterm., Reinwardtia 6(3): 279. 1962. TYPE: MEXICO. Tabasco: Lomas de San Sebastián, 26 Mar 1889 (fl), *Rovirosa 416* (holotype: K; isotypes: F, NY! [00546807], US! [00098426]).

#### Distribution.

Southern Mexico to Costa Rica, and in western Cuba. A collection stated to be from St. Lucia (the type of Belotia caribaea) represents either material cultivated in the St. Vincent Botanic Garden or mislabeled material from Cuba (Bornstein 1989: 185–186).

#### Note.

The name Trichospermum grewiifolium (A. Rich.) Kosterm. is frequently applied to this species, but it is illegitimate because as explained in the text Belotia grewiifolia A. Rich. was nomenclaturally superfluous when published (McNeill et al. 2006: Art. 52.1) and cannot serve as the basionym for this combination. More recently, Rodríguez Fuentes (2000: 32) accepted Trichospermum grewiifolium as a new species published by Kostermans (1962) apparently in the belief that Kostermans had explicitly excluded the purported basionym’s type, but this is debatable and in any case Trichospermum grewiifolium is not a valid name as Kostermans failed to designate a nomenclatural type (McNeill et al. 2006: Art. 37.1).

### Trichospermum mexicanum

(DC.)

Trichospermum mexicanum  (DC.) Baill., Hist. Pl. 4: 179. 1872 (excluding synonym Adenodiscus mexicanus Turcz.). Grewia mexicana DC., Prodr. 1: 510. 1824. Belotia mexicana (DC.) K. Schum. in Engler & Prantl, Nat. Pflanzenfam. 3(6): 28. 1890. Belotia grewiifolia A. Rich. in R. de la Sagra, Hist. Phys. Cuba, Pl. Vasc.: 209, t. 21. 1841 [1845], as “*greviæfolia*,” nom. illeg. TYPE: MEXICO. “Nova Hispania,” 1807 (fl), *Lagasca y Segura 86* (holotype: G-DC [IDC microfiche 216!]).
Belotia insignis  Baill., Adansonia 10: 182. 1872. Trichospermum insigne (Baill.) Kosterm., Reinwardtia 6(3): 279. 1962, as “*insignis*.” TYPE: MEXICO. “Andes of Mexico,” *Ghiesbreght 356* (holotype: P, photo [F neg. no. 35430] US!; isotype: F).

#### Distribution.

Endemic to western and southern Mexico, where it appears to be restricted to the Pacific lowlands and hills from Sinaloa to Oaxaca.

#### Note.

McVaugh (2000: 526–527) speculated that the type of Grewia mexicana may have been a garden specimen grown at Madrid from Mexican seed contributed by the Expedición Real.

## Supplementary Material

XML Treatment for Trichospermum lessertianum

XML Treatment for Trichospermum mexicanum
